# Comparing Cardiac Reverse Remodeling in Aortic Stenosis With Surgical and Transcatheter Aortic Valve Replacement

**DOI:** 10.1016/j.atssr.2025.03.019

**Published:** 2025-04-15

**Authors:** Koichi Inoue, Koichi Maeda, Kyongsun Pak, Kazuo Shimamura, Arudo Hiraoka, Hidenori Yoshitaka, Katsukiyo Kitabayashi, Haruhiko Kondoh, Yukitoshi Sirakawa, Shigeru Miyagawa

**Affiliations:** 1 Department of Cardiovascular Surgery, Osaka University Graduate School of Medicine, Osaka, Japan; 2 Division of Biostatistics, Center for Clinical Research, National Center for Child Health and Development, Tokyo, Japan; 3 Department of Cardiovascular Surgery, The Sakakibara Heart Institute of Okayama, Okayama, Japan; 4 Department of Cardiovascular Surgery, Japan Organization of Occupational Health and Safety Kansai Rosai Hospital, Hyogo, Japan; 5 Department of Cardiovascular Surgery, Japan Organization of Occupational Health and Safety Osaka Rosai Hospital, Osaka, Japan; 6 Department of Cardiovascular Surgery, Osaka General Medical Center, Osaka, Japan

## Abstract

**Background:**

Left ventricular reverse remodeling, which is synonymous left ventricular mass regression, after surgical aortic valve replacement (SAVR) or transcatheter aortic valve replacement (TAVR) is associated with positive clinical outcomes in patients with aortic stenosis. However, the roles of SAVR and TAVR in left ventricular mass regression remain unclear. This study compared the left ventricular mass change between SAVR and TAVR.

**Methods:**

Included were 1939 patients with aortic stenosis who underwent isolated SAVR or TAVR, and 1:1 propensity matching was performed (247 pairs). The primary outcome was the time course change of left ventricular mass between SAVR and TAVR. Left ventricular mass regression was evaluated and calculated up to 1 year of follow-up by echocardiography.

**Results:**

In a matched cohort, SAVR demonstrated better left ventricular mass regression compared with TAVR at 30 days (SAVR vs TAVR: mean, −11.2% [95% CI, −13.4% to −8.9%] vs mean, −2.6% [95% CI, −5.0% to −0.4%], *P* < .01) and at 1 year (SAVR vs TAVR: mean −23.8% [95% CI, − 26.0% to −21.6%) vs −13.8% [95% CI, −16.6% to −11.0%], *P* < .01). In multivariable analysis, baseline left ventricular mass index (odds ratio, 1.04; 95% CI, 1.03-1.05; *P* < .01), SAVR choice (odds ratio, 2.54; 95% CI, 1.46-4.43; *P* < .01), and paravalvular leakage mild or more (odds ratio, 0.61; 95% CI, 0.44-0.84; *P* < .01) were associated with left ventricular mass regression.

**Conclusions:**

SAVR demonstrated better left ventricular mass regression than TAVR in a matched cohort. Considering the lifetime management of the patients, selecting the optimal valve is crucial.

## Visual Abstract

**Figure:**
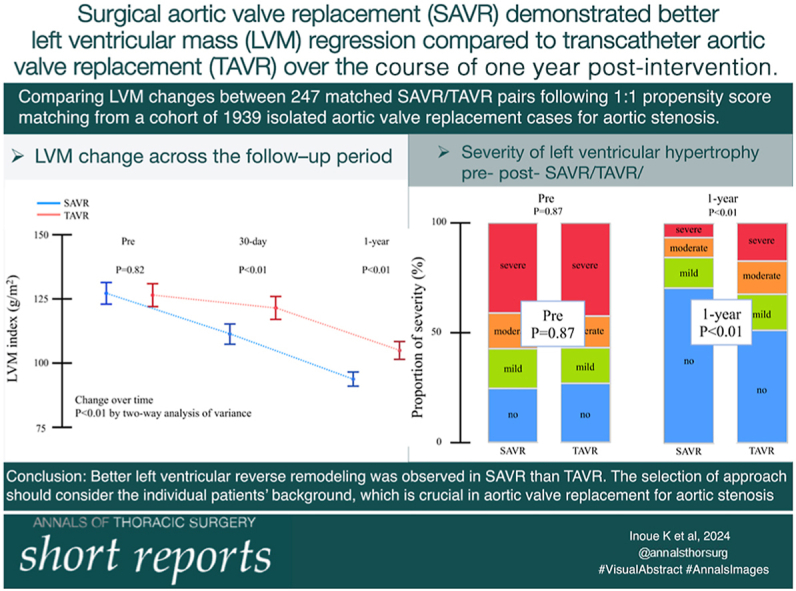

**In Short**

▪ In patients with aortic stenosis, surgical aortic valve replacement demonstrated superior left ventricular mass regression compared with transcatheter aortic valve replacement in a matched cohort.
▪ The choice of valve intervention and the presence of paravalvular leakage were identified as pivotal and modifiable factors influencing left ventricular mass regression after aortic valve replacement.

Aortic stenosis (AS) is a common heart valvular disease worldwide that significantly reduces the patients’ quality of life and survival.^1^ Left ventricular hypertrophy (LVH) commonly manifests in patients with AS owing to prolonged pressure overload to the cardiac muscle and is significantly correlated to AS progression. Aortic valve replacement is the gold standard method of treatment for AS. Unloading the AS-afflicted heart through valve replacement prompts left ventricular reverse remodeling, synonymous with left ventricular mass (LVM) regression. Moreover, LVM regression subsequent to intervention is considered to have a positive impact on clinical outcomes.^2^

In the past decade, transcatheter aortic valve replacement (TAVR) has demonstrated substantial advantages over surgical aortic valve replacement (SAVR).^3^ TAVR is also believed to lead as well as SAVR to LVM regression. However, whether SAVR or TAVR is superior in inducing LVM regression remains unclear. In this study, we performed a comparative analysis of LVM alterations over time after SAVR and TAVR in a propensity-matched cohort. This investigation has significant implications for reassessing patient selection criteria for AS interventions.

## Patients and Methods

### Study Design and Patient Selection

This study is a retrospective, observational analysis derived from the Accelerating Conventional and Transcatheter Integration in Valvular Intervention Strategy (ACTIVIST) registry from Japan. Of 2492 patients who were included, 1939 patients underwent SAVR (n = 490) or TAVR (n = 1449) between April 2016 and December 2021. Candidacy for SAVR or TAVR was determined based on Japanese guideline recommendations through a heart team discussion. The details of interventional indications, valves used, echocardiographic measurement, and statistical analysis are shown in the Supplemental Materials and Supplemental Table 1.

### Study Approval and Informed Consent

The Osaka University Medical Hospital Institutional Review Board of (Clinical trial No. 20222(T2); date, 2021/12/15) approved this study, and the patients gave written informed consent.

### Outcomes

The primary outcome encompassed the LVM index change from baseline to the 1-year follow-up, along with the proportion of patients exhibiting LVM regression after SAVR or TAVR. Key factors associated with LVM regression were analyzed across the entire cohort. Secondary outcomes included clinical outcomes, such as all-cause mortality, cardiac-related mortality, and major adverse cardiovascular and cerebrovascular events. Clinical outcomes are defined in the Supplemental Materials.

## Results

### Patient Demographics

A total of 247 patients who underwent SAVR were propensity score matched 1:1 with 247 patients who underwent TAVR during the corresponding period. The characteristics of all patients (n = 1939) and the propensity-matched cohort are summarized in Supplemental Table 2.

The postoperative findings in the matched cohort are presented in Supplemental Table 3. Postoperative echocardiography revealed better LVM regression in the SAVR group. Implanted valve peak velocity was significantly higher in the SAVR group, with a greater prevalence of prosthetic-patient mismatch (PPM) in SAVR than in TAVR (66 [26.8%] vs 45 [18.2%] patients, *P* < .05). Paravalvular leakage (PVL) was significantly lower in the SAVR group than in the TAVR group (5 [2.0%] vs 89 [36.0%] patients, *P* < .01).

### Comparison of LVM Change Between SAVR and TAVR

**Figure 1:**
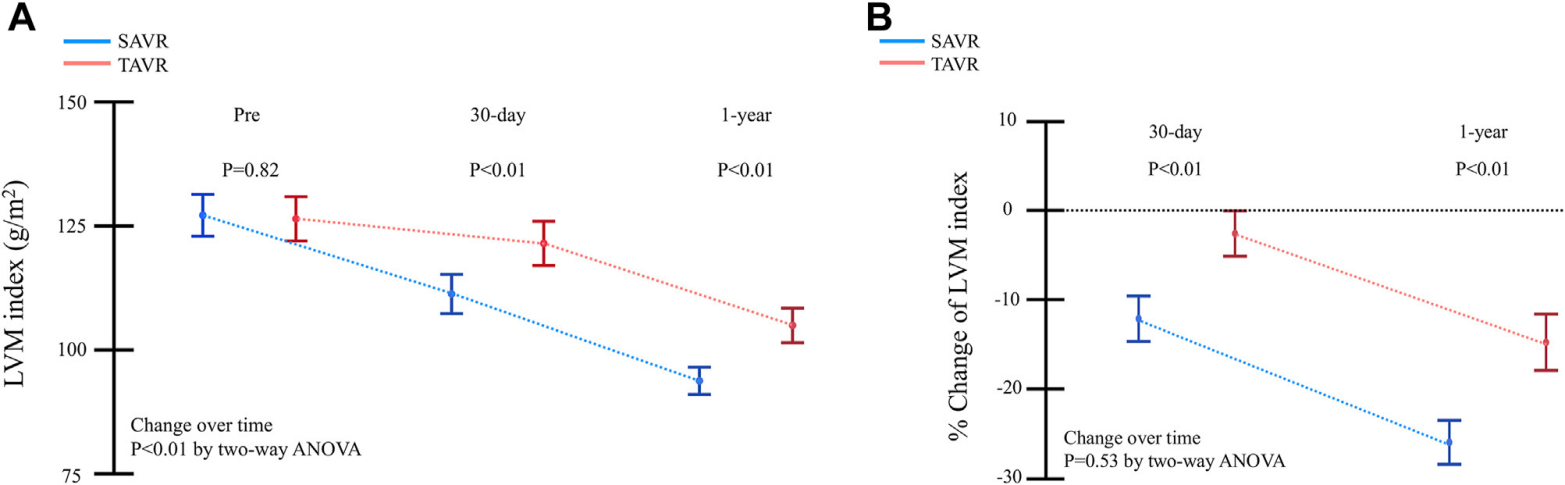
Actual left ventricular mass (LVM) change at baseline and at serial times after surgical aortic valve replacement (SAVR) and transcatheter aortic valve replacement (TAVR). (A) Comparison of absolute changes in LVM index from baseline. (B) Comparison of percentage of LVM index changes from baseline. (ANOVA, analysis of variance.)

**Figure 2:**
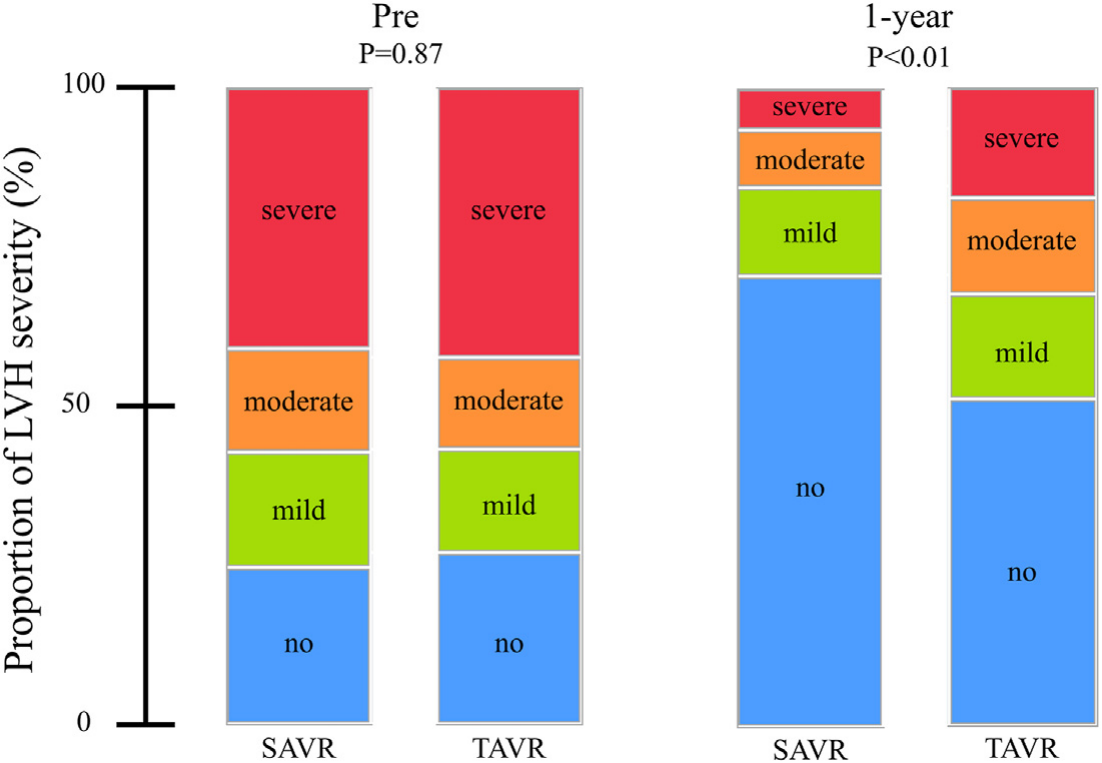
Proportional difference in left ventricular hypertrophy (LVH) severity at baseline and at the 1-year follow-up after surgical aortic valve replacement (SAVR) and transcatheter aortic valve replacement (TAVR).

In the propensity-matched cohort, SAVR demonstrated reduced LVM index at the 30-day (SAVR vs TAVR: mean, 110.6 g/m^2^ [95% CI, 106.6-114.5 g/m^2^] vs mean, 120.7 g/m^2^ [95% CI, 116.3-125.2 g/m^2^]; *P* < .01) and 1-year (SAVR vs TAVR: mean, 93.1 g/m^2^ [95% CI, 90.4-95.9 g/m^2^] vs mean, 104.2 g/m^2^ [95% CI, 100.8-107.7 g/m^2^]; *P* < .01) follow-up echocardiography assessments (Figure 1A). Although LVM regression from the baseline was evident in a substantial number of patients in both groups, the magnitude of regression was significantly greater in the SAVR group at 30 days (SAVR vs TAVR: mean, −11.2% [95% CI, −13.4% to −8.9%] vs mean, −2.6% [95% CI, −5.0% to −0.4%]; *P* < .01) and 1 year (SAVR vs TAVR: mean −23.8% [95% CI, −26.0% to −21.6%] vs mean, −13.8% [95% CI, −16.6% to −11.0%]; *P* < .01) (Figure 1B). At the 1-year follow-up, ∼70% of patients exhibited restoration to a normal ventricular state in the SAVR group, whereas, nearly half of the patients in the TAVR group demonstrated absence of LVH, with moderate or severe LVH persisting in ∼30% of patients (Figure 2).

### Factors for LVM Regression

Table 1 delineates the outcomes of the multivariable analysis using a nominal logistic regression model. Baseline LVM index (odds ratio [OR], 1.04; 95% CI, 1.03-1.05; *P* < .01), left ventricular systolic diameter (LVDs) (OR, 0.87; 95% CI, 0.79-0.95; *P* < .01), left atrium diameter (LAD) index (OR, 0.97; 95% CI, 0.94-0.99; *P* = .04), SAVR choice (OR, 2.54; 95% CI, 1.46–4.43; *P* < .01), and the presence of mild or greater PVL (OR, 0.61; 95% CI, 0.44–0.84; *P* < .01) emerged as independently associated factors for LVM regression. In summary, a higher baseline LVM index and smaller LVDs, LAD index, and SAVR choice were positively correlated with LVM regression, whereas the presence of mild or greater PVL exerted a negative influence.

**Table:** Variables Associated With LVM Regression at 1 Year After Intervention

Variables	Univariable Model		Multivariable Model	
	OR for LVM Regression (95% CI)	*P* Value	OR for LVM Regression (95% CI)	*P* Value
Age (y)	0.98 (0.96-0.99)	.01	1.01 (0.98-1.04)	.39
Female sex	0.82 (0.65-1.03)	.09	0.81 (0.59-1.11)	.19
STS-PROM (%)	0.97 (0.94-0.99)	.01	0.98 (0.94-1.03)	.41
Diabetes mellitus	0.94 (0.74-1.20)	.62	1.03 (0.74-1.43)	.86
Atrial fibrillation	0.78 (0.57-1.07)	.12	1.25 (0.82-1.92)	.30
Peripheral arterial disease	0.85 (0.59-1.24)	.41	1.09 (0.66-1.79)	.75
Bicuspid aortic valve	1.60 (1.03-2.50)	.04	1.10 (056-2.14)	.79
Creatinine (mg/dL)	1.06 (1.14-0.94)	.09	0.92 (0.83-1.02)	.14
Left ventricular diameter				
Diastolic (mm)	1.07 (1.05-1.09)	<.01	1.06 (0.99-1.13)	.09
Systolic (mm)	1.04 (1.02-1.06)	<.01	0.87 (0.79-0.95)	**<.01**
LAD index (mm/m^2^)	1.00 (0.98-1.02)	.87	0.97 (0.94-0.99)	**.04**
Left ventricular				
Ejection fraction	0.99 (0.99-1.00)	.24	0.97 (0.94-1.00)	.05
Mass index (g/m^2^)	1.03 (1.03-1.04)	<.01	1.04 (1.03-1.05)	**<.01**
Aortic insufficiency grade	1.14 (1.01-1.28)	.03	0.98 (0.84-1.16)	.83
TRPG (mm Hg)	1.01 (0.99-1.02)	.31	1.01 (0.99-1.02)	.37
Aortic valve				
Peak velocity (m/s)	1.96 (1.65-2.32)	<.01	1.08 (0.61-1.89)	0.79
Mean PG (mm Hg)	1.03 (1.02-1.04)	<.01	1.00 (0.98-1.03)	0.54
SAVR	2.98 (2.12-4.19)	<.01	2.54 (1.46-4.43)	**<.01**
PVL ≥mild	0.73 (0.58-0.92)	<.01	0.61 (0.44-0.84)	**<.01**
PPM ≥moderate	1.24 (0.91-1.69)	.18	1.28 (0.84-1.96)	.24
New pacemaker implant	0.58 (0.41-0.81)	<.01	0.77 (0.49-1.23)	.28

Bold *P* values indicate statistical significance (*P* < .05).

LAD, left atrium diameter; LVM, left ventricular mass; OR, odds ratio; PG, pressure gradient; PPM, prosthesis-patients mismatch; PVL, paravalvular leakage; SAVR, surgical aortic valve replacement; STS-PROM; Society of Thoracic Surgery-Predicted Risk of Mortality; TRPG, tricuspid regurgitant pressure gradient.

### Comparison of Clinical Outcomes

The comparison of clinical outcomes is illustrated in Supplemental Figures 1 and 2. In the matched cohort, SAVR demonstrated superior overall survival and freedom from cardiac death at the 5-year follow-up.

## Comment

In summary, SAVR demonstrated better LVM regression than TAVR from baseline to the 1-year follow-up in the propensity-matched cohort, and the baseline values of LVM index, LVDs, and LAD index, along with SAVR choice and the presence of PVL, were independently correlated with LVM regression.

LVH develops in patients with AS precipitating pathologic adaptations in the left ventricle as a response to increased blood pressure. LVM regression, a phenomenon anticipated after the alleviation of pressure overload postintervention for AS, signifies left ventricular reverse remodeling and strongly influences long-term clinical outcomes.^2^ Therefore, interventions should be considered to avoid missing the window of the reversible phase of LVH, with preference given to interventions fostering LVM regression when feasible.

TAVR has yielded satisfactory short- and midterm outcomes for patients with AS.^3^ However, the long-term clinical outcomes and the expansion of TAVR indications remain contentious. In our study, SAVR demonstrated greater LVM regression than TAVR, and SAVR itself had a positive impact for LVM regression. This difference is likely due to the prosthetic valve features between SAVR and TAVR.

**Supplementary Figure 3:**
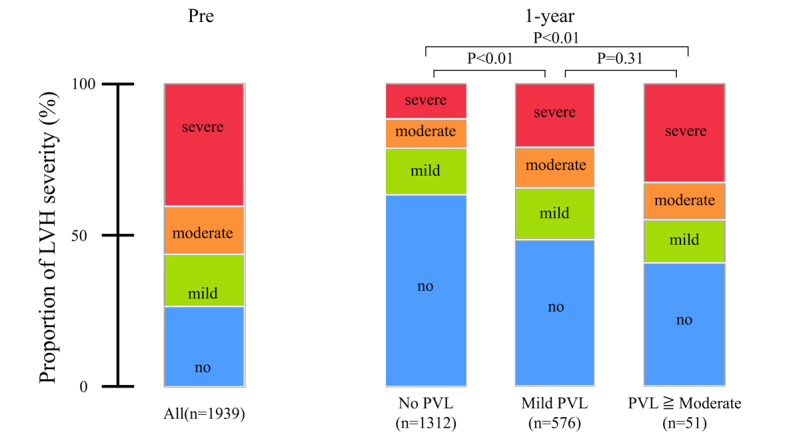


In this study, we focused on residual PVL. PVL is one of the foremost concerns associated with TAVR. Even mild PVL is a well-known negative factor for clinical outcomes.^4^ Industries continue to make efforts to reduce PVL; however, even with the latest generation transcatheter heart valve, PVL remains present in >15% of cases.^5^ In this cohort, PVL emerged as one of the essential factors for LVM regression and may be considered one of the factors that inhibits LVM regression by SAVR or TAVR selection (Supplemental Table 4). The severity of PVL influences LVM regression (Supplemental Figure 3), and even mild PVL has a substantial influence on LVM regression.

PPM, especially if it is severe, is also reported to be associated with higher mortality after TAVR.^6^ Although a direct comparison is not available, a review of previous systematic studies suggests that PVL has a greater impact on postoperative prognosis after aortic valve replacement in hazard ratio compared with PPM.^5^^,^^6^ Furthermore, PPM was observed in a certain proportion of both TAVR and SAVR patients in this study. However, PVL was rarely detected in SAVR. Therefore, when considering long-term prognosis, adopting a strategy to minimize PVL appears to be a reasonable approach.

A definitive understanding of the factors influencing long-term prognosis when selecting a treatment strategy for AS remains unresolved. However, with advancements in AS treatment outcomes and a growing emphasis on improving patients’ quality of life beyond survival, considering consider treatment options that promote cardiac reverse remodeling in patients with a favorable long-term prognosis is essential.

### Study Limitations

This study was a descriptive retrospective analysis of registry data and was not hypothesis driven. Although propensity score matching was performed, it remains possible that there may have been residual differences in the SAVR and TAVR populations. Furthermore, our assessment of LVM change was limited to echocardiography up to the 1-year follow-up, and there was a lack of data beyond the 1-year follow-up. Consequently, there is a possibility that any observed differences including LVM regression, PVL, or PPM may diminish or widen over time.

### Conclusions

In this study, SAVR exhibited better LVM regression than TAVR in the propensity-matched patients with AS. An extensive baseline LVM index was strongly correlated with LVM regression, consistent with previous findings. Remarkably, SAVR/TAVR valve choice and PVL were preventable factors contributing to enhanced LVM regression. This underscores the pivotal consideration of tailored patient selection in aortic valve replacement interventions.

## Figures and Tables

**Supplementary Figure 1A:**
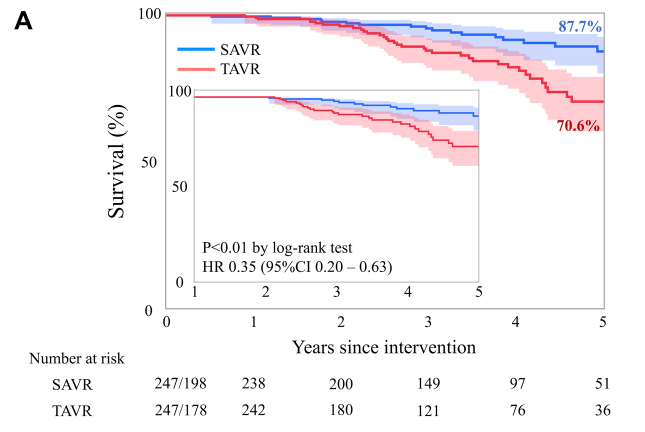


**Supplementary Figure 1B:**
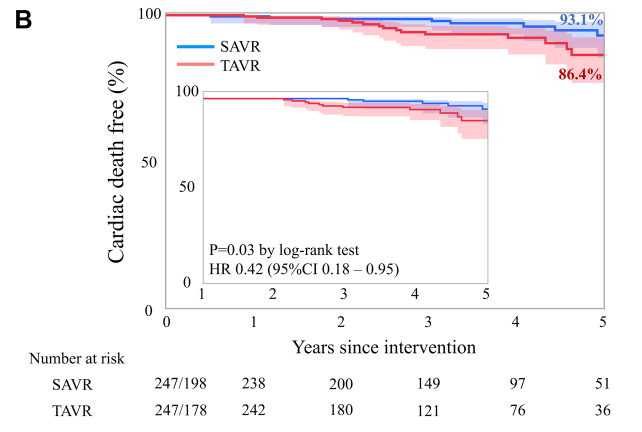


**Supplementary Figure 2A:**
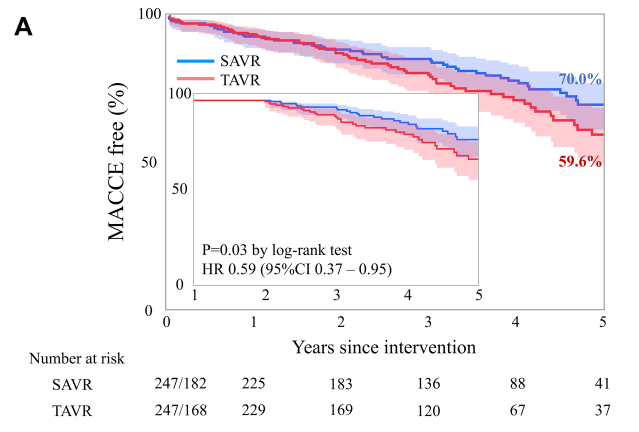


**Supplementary Figure 2B:**
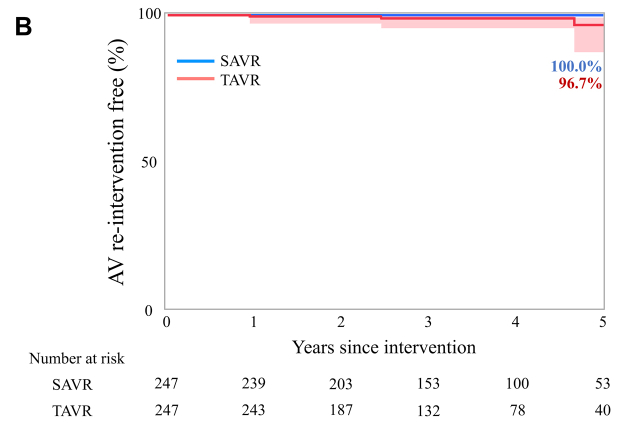


**Supplementary Figure 2C:**
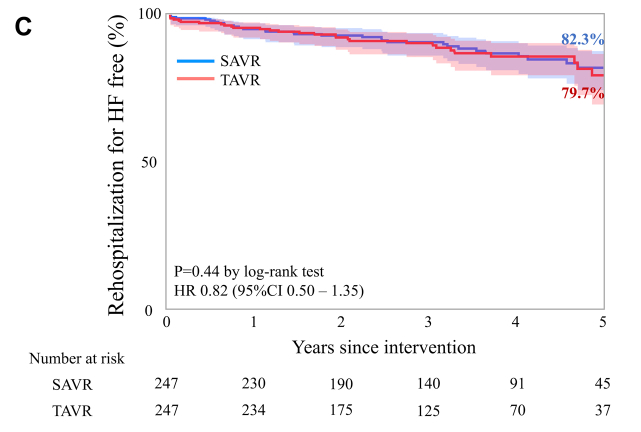


**Supplementary Figure 2D:**
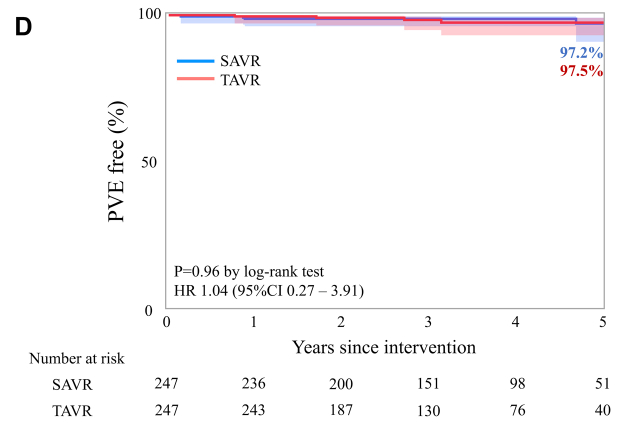


**Supplementary Figure 2E:**
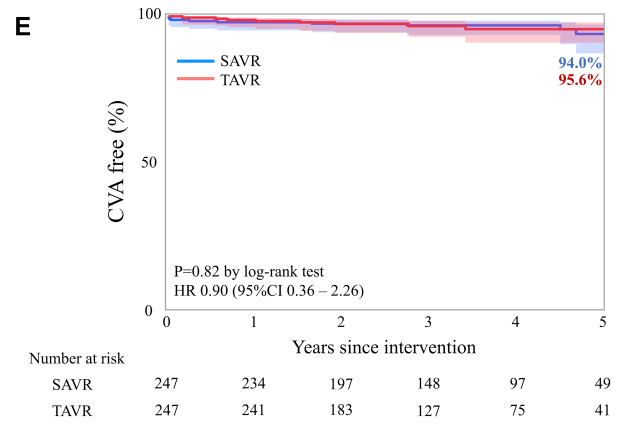


**Supplementary Figure 2F:**
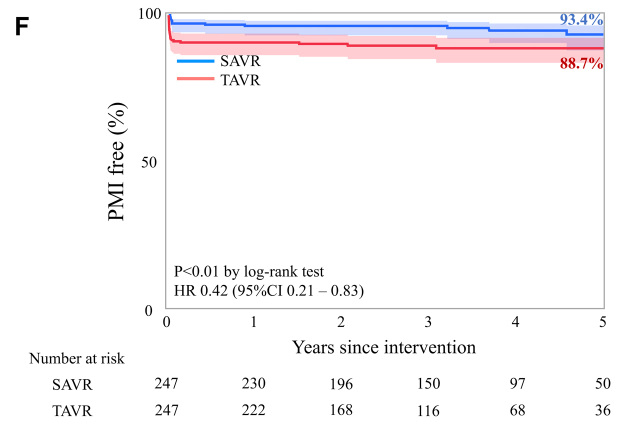


## References

[1] Carabello B.A., Paulus W.J. (2009). Aortic stenosis. Lancet.

[2] Ali A., Patel A., Ali Z. (2011). Enhanced left ventricular mass regression after aortic valve replacement in patients with aortic stenosis is associated with improved long-term survival. J Thorac Cardiovasc Surg.

[3] Leon M.B., Smith C.R., Mack M.J. (2016). Transcatheter or surgical aortic-valve replacement in intermediate-risk patients. N Engl J Med.

[4] Sá M.P., Jacquemyn X., Van den Eynde J. (2022). Impact of paravalvular leak on outcomes after transcatheter aortic valve implantation: meta-analysis of Kaplan-Meier-derived individual patient data. Struct Heart.

[5] Nazif T.M., Cahill T.J., Daniels D. (2021). Real-world experience with the SAPIEN 3 Ultra transcatheter heart valve: a propensity-matched analysis from the United States. Circ Cardiovasc Interv.

[6] Sá M.P., Jacquemyn X., Van den Eynde J. (2023). Impact of prosthesis-patient mismatch after transcatheter aortic valve replacement: meta-analysis of Kaplan-Meier-derived individual patient data. JACC Cardiovasc Imaging.

