# Supplementary material for: Comparing Cardiac Reverse Remodeling in Aortic Stenosis With Surgical and Transcatheter Aortic Valve Replacement
Source: Ann Thorac Surg Short Rep. 2025 Apr 15;3(3):624–8. doi: 10.1016/j.atssr.2025.03.019 (PMC12559588; doi:10.1016/j.atssr.2025.03.019)
Supplement: Supplementary Material [file mmc1.docx]

**Patients and Methods**

This study is a retrospective, observational analysis derived from Accelerating Conventional and Transcatheter Integration in Valvular Intervention Strategy: ACTIVIST registry from Japan. A total of 1939 patients who underwent aortic valve replacement (SAVR, n=490; TAVR, n=1449) between April 2016 and December 2021 out of 2492 patients were included. Patients who had no echocardiogram records at 1-year follow-up and underwent concomitant surgeries of the coronary arteries, aorta, or other valves were excluded. Interventional indications were predicated on the severity of AS or heart failure deemed attributable to AS, at the discretion of the cardiologist. TAVR eligibility was determined based on guideline recommendations for individuals age ≥ 80 years or those with intermediate or high STS-PROM scores. Conversely, SAVR candidacy was principally reserved for younger patients (aged <80 years) and those with low STS-PROM scores. However, patients with anatomically complex cases, such as those with narrow access routes, a shaggy aorta or a horizontal aorta, who were deemed unsuitable for TAVR, or those who preferred surgery, underwent SAVR as determined by the heart team discussion. Severe AS was defined as an aortic valve area <1.0cm^2^ (or indexed aortic valve area <0.6 cm^2^/m^2^) and either resting or inducible aortic valve mean gradient ≥ 40 mmHg or peak velocity ≥ 4 m/s. LVH categories were defined based on sex-specific thresholds of LVM index.^1^ The types of valves were selected by guideline recommendation or institute’s and surgeon’s preference. Bioprosthetic, mechanical and sutureless valves were used in the SAVR group and balloon-expandable and self-expandable valves were in the TAVR group.

**Echocardiography**

Transthoracic echocardiographic assessments were conducted at baseline and at 30-day and 1-year intervals after the intervention. LVM was calculated using the formula recommended by the American Society of Echocardiography.^1^ LVM regression was defined as a negative percentage change in LVM index from baseline to 1-year post-intervention. Paravalvular leakage (PVL) was determined and graded using a multiparameter integrative methodology.^2^ In instances where the peak velocity or mean pressure gradient of the implanted valve was significantly augmented, prosthetic-patient mismatch (PPM) was calculated as the ratio of the aortic valve area to the body surface area. A value of <0.85 cm^2^/m^2^ was defined as moderate or greater PPM.^3^

**Outcomes**

Cardiac death was defined as death due to myocardial infarction, heart failure, arrhythmia or sudden death. MACCE included events of all-cause mortality, re-intervention on the aortic valve, rehospitalization due to heart failure, prosthetic valve endocarditis, and cerebrovascular accidents. All events were collected and identified through electronic medical records.

**Statistical Analysis**

Continuous data were presented as the mean and 95%CI if they followed a normal distribution, and the median and interquartile values (1st quartile to 3rd quartile) if they did not. Continuous data were compared using Student’s t-test if they followed a normal distribution, and the Mann–Whitney U test if they did not. Categorical variables were expressed as a counts and percentages of patients, and comparisons were made using the χ^2^ test. Fisher’s exact test was employed when any expected frequency fell below 1, or when 20% of expected frequencies were ≤ 5. Statistical significance was set at P-value <0.05. To compare patients who underwent SAVR and TAVR, 1:1 propensity score and nearest neighbor matching without replacement were executed, employing a caliper of 0.2 standard deviation (logit) for the multivariable logistic regression model. The model designated SAVR/TAVR as the objective variable, with explanatory variables including age, female sex, STS-PROM, hypertension, serum hemoglobin, serum albumin, bicuspid aortic valve, baseline left ventricular diastolic diameter, left ventricular systolic diameter, left ventricular ejection fraction, left atrial diameter index, LVM index, and aortic valve peak velocity. Additionally, the balance of the baseline characteristic distribution between the two groups was assessed by evaluating the standardized mean difference (SMD) which less than 0.1 was considered the acceptable range. Differences in the trajectory of LVM changes across the follow-up time course at baseline, 30-day, and 1-year after intervention were analyzed using two-way repeated measures analysis of variance (ANOVA) with group and time as factors. Time-to-event outcomes were evaluated using the Kaplan-Meier method, Log-rank test and Cox regression analysis. Additionally, for the outcomes did not adhere to the proportional hazard assumption, a landmark analysis was conducted using only cases from 2-year follow-up onward. The key determinants influencing the LVM regression were scrutinized from the entire cohort using a multi variable nominal logistic regression model. We defined LVM regression as a categorical variable according to negative or positive LVMi change from baseline to 1-year follow-up. 21 variables were extracted for a multi variable analysis based on the criteria that they had a statistical relevance to LVM regression in univariate analysis (P < 0.05), were reported to be related to LVM regression in previous literature, or were considered clinically important.^4-7^ All data analyses were conducted using the Statistical Analysis Systems software JMP Pro 17 (SAS Institute Inc. Cary, NC, USA).

**References**

[1] Lang RM, Bierig M, Devereux RB, et al. Recommendations for chamber quantification: a report from the American Society of Echocardiography’s Guidelines and Standards Committee and the Chamber Quantification Writing Group, developed in conjunction with the European Association of Echocardiography, a branch of the European Society of Cardiology. *J Am Soc Echocardiogr*. 2005; 18(12): 1440-1463.

[2] Pibarot P, Hahn RT, Weissman NJ, Monaghan MJ. Assessment of paravalvular regurgitation following TAVR: a proposal of unifying grading scheme. *JACC Cardiovasc Imaging*. 2015; 8(3): 340-360.

[3] Pibarot P, Dumesnil JG. Prosthesis-patient mismatch: definition, clinical impact, and prevention. *Heart*. 2006; 92(8): 1022-1029.

[4] Ali A, Patel A, Ali Z, et al. Enhanced left ventricular mass regression after aortic valve replacement in patients with aortic stenosis is associated with improved long-term survival. *J Thorac Cardiovasc Surg*. 2011; 142(2):285-291.

[5] Chau KH, Douglas PS, Pibarot P, et al. Regression of left ventricular mass after transcatheter aortic valve replacement: The PARTNER trials and registries. *J Am Coll Cardiol*. 2020; 75(19): 2446-2458.

[6] Nakamura T, Toda K, Kuratani T, et al. Diabetes mellitus impairs left ventricular mass regression after surgical or transcatheter aortic valve replacement for severe aortic stenosis. *Heart Lung Circ*. 2016; 25(1):68-74.

[7] Hachiro K, Kinoshita T, Asai T, Suzuki T. Left ventricular mass regression in patients without patient-prosthesis mismatch after aortic valve replacement for aortic stenosis. *Gen Thorac Cardiovasc Surg*. 2020; 68(3). 227-232.

**Supplemental Table 1. Implanted valve details in matched cohort.**

|  | **Propensity-matched patients** | |
| --- | --- | --- |
| **Variables** | **SAVR**  **(n=247)** | **TAVR**  **(n=247)** |
| **Implanted valve product, n** |  |  |
| **Edwards Lifesciences (Irvine, California, U.S.)** |  |  |
| **Magna/Magna EASE** | 124 (50.2%) |  |
| **Inspiris** | 38 (15.4%) |  |
| **Intuity** | 15 (6.7%) |  |
| **Sapien XT** |  | 7 (2.8%) |
| **Sapien 3** |  | 143 (57.9%) |
| **Medtronic (**Dublin, Ireland) |  |  |
| **Avalus** | 3 (1.2%) |  |
| **Mosaic/Mosaic Ultra** | 12 (4.9%) |  |
| **CoreValve** |  | 31 (12.6%) |
| **Evolut R** |  | 31 (12.6%) |
| **Evolut Pro/Pro plus** |  | 34 (13.8%) |
| **Abbott / St Jude Medical (North Chicago, Illinois, U.S.)** |  |  |
| **Trifecta** | 5 (20.2%) |  |
| **Epic** | 2 (0.8%) | - |
| **SJM** | 1 (0.4%) |  |
| **Portico** |  | 1 (0.4%) |
| **LivaNova (London, U.K.)** |  |  |
| **Mitroflow** | 7 (2.8%) |  |
| **Perceval** | 34 (13.8%) |  |
| **Artivion (Kennesaw, Georgia, U.S.)** |  |  |
| **On-X** | 6 (2.4%) |  |
| **Implanted valve size, n** |  |  |
| **17mm** | 1 (0.4%) | - |
| **19mm** | 49 (19.8%) | - |
| **20mm** | 1 (0.4%) | 10 (4.1%) |
| **21mm** | 118 (47.8%) | - |
| **23mm** | 52 (21.1%) | 83 (33.6%) |
| **24mm** | 1 (0.4%) | - |
| **25mm** | 23 (9.3%) | 1 (0.4%) |
| **26mm** | - | 95 (38.5%) |
| **27mm** | 2 (0.8%) | - |
| **29mm** | - | 55 (22.3%) |
| **34mm** | - | 3 (1.2%) |

SAVR, surgical aortic valve replacement; TAVR, transcatheter aortic valve replacement**.**

**Supplemental Table 2. Patients’ baseline demographics**

|  | **All patients** | |  | **Propensity-matched patients** | |  |
| --- | --- | --- | --- | --- | --- | --- |
| **Variables** | **SAVR (n=490)** | **TAVR (n=1449)** | **SMD** | **SAVR (n=247)** | **TAVR (n=247)** | **SMD** |
| **Age, years**** | 75 [70 – 79] | 84 [81 – 87] | 1.49 | 79 [76 – 81] | 79 [75 – 82] | <0.1 |
| **Female, n** | 263 (53.7%) | 860 (59.4%) | 0.12 | 145 (58.7%) | 145 (58.7%) | <0.1 |
| **BSA, m^2^*** | 1.57(1.55 – 1.59) | 1.45 (1.44 – 1.46) | 0.68 | 1.53 (1.51 – 1.55) | 1.54 (1.52 – 1.56) | <0.1 |
| **BMI, kg/m^2^*** | 23.7 (23.3 – 24.0) | 22.5 (22.3 – 22.7) | 0.32 | 23.3 (22.8 – 23.7) | 23.4 (22.9 – 23.8) | <0.1 |
| **STS-PROM score, %**** | 2.4 [1.4 – 3.7] | 5.1 [3.8 – 7.6] | 0.98 | 3.14 [2.15 – 4.56] | 3.57 [2.56 – 4.73] | <0.1 |
| **Comorbidities** |  |  |  |  |  |  |
| **Hypertension, n** | 352 (71.8%) | 1115 (77.0%) | 0.12 | 187 (75.7%) | 188 (75.9%) | <0.1 |
| **Dyslipidemia, n** | 258 (52.7%) | 721 (49.8%) | 0.06 | 127 (51.4) | 127 (51.4) | <0.1 |
| **Diabetes mellitus, n** | 134 (27.4%) | 417 (28.8%) | 0.03 | 74 (30.0%) | 79 (32.0%) | <0.1 |
| **CKD, n** | 6140 (28.6%) | 575 (39.7%) | 0.23 | 83 (33.6%) | 93 (37.7%) | <0.1 |
| **Hemodialysis, n** | 56 (11.4%) | 70 (4.8%) | 0.27 | 29 (11.7%) | 19 (7.7%) | 0.13 |
| **Smoking, n** | 77 (15.7%) | 118 (8.1%) | 0.25 | 30 (12.2%) | 37 (15.0%) | <0.1 |
| **Atrial fibrillation, n** | 53 (10.8%) | 208 (14.4%) | 0.10 | 27 (12.2%) | 21 (9.5%) | <0.1 |
| **PMI, n** | 14 (2.9%) | 78 (5.4%) | 0.12 | 7 (3.2%) | 9 (4.1%) | <0.1 |
| **OMI, n** | 9 (1.8%) | 58 (4.0%) | 0.12 | 4 (1.8%) | 5 (2.3%) | <0.1 |
| **PAD, n** | 33 (6.7%) | 143 (9.9%) | 0.11 | 19 (8.6%) | 21 (9.5%) | <0.1 |
| **BAV, n** | 121 (24.7%) | 53 (3.7%) | 0.74 | 25 (10.1%) | 23 (9.3%) | <0.1 |
| **Laboratory data** |  |  |  |  |  |  |
| **Hemoglobin, g/dl*** | 12.5 (12.4 – 12.7) | 11.4 (11.4 – 11.6) | 0.62 | 12.1 (11.9 – 12.3) | 12.0 (11.8 – 12.3) | <0.1 |
| **Albumin, g/dl*** | 3.9 (3.9 – 4.0) | 3.7 (3.7 – 3.8) | 0.47 | 3.9 (3.8 – 3.9) | 3.8 (3.8 – 3.9) | <0.1 |
| **Creatinine, mg/dl**** | 0.85 [0.69 – 1.07] | 0.90 [0.72 – 1.21] | 0.22 | 0.88 [0.72 – 1.18] | 0.90 [0.71 – 1.20] | 0.12 |
| **Echocardiography** |  |  |  |  |  |  |
| **LVDd, mm*** | 47.0 (46.4 – 47.5) | 44.9 (44.6 – 45.3) | 0.31 | 46.1 (45.4 – 46.8) | 46.0 (45.1 – 46.8) | <0.1 |
| **LVDs, mm*** | 29.9 (29.3 – 30.5) | 29.5 (29.1 – 29.9) | 0.06 | 29.5 (28.7 – 30.3) | 29.3 (28.4 – 30.2) | <0.1 |
| **LADi, mm/m^2^*** | 26.8 (26.4 – 27.3) | 30.2 (30.0 – 30.5) | 0.64 | 28.0 (27.4 – 28.7) | 27.8 (27.2 – 28.4) | <0.1 |
| **LVEF, %**** | 68 [61 – 73] | 66 [59 – 72] | 0.19 | 68 [60 – 73] | 67 [61 – 74] | <0.1 |
| AI ≥ moderate, n | 80 (16.3%) | 194 (13.4%) | 0.08 | 32 (13.0%) | 31 (12.6%) | <0.1 |
| **MR ≥** **moderate, n** | 32 (6.5%) | 174 (12.0%) | 0.18 | 21 (8.5%) | 20 (8.1%) | <0.1 |
| **TR ≥** **moderate, n** | 11 (2.2%) | 117 (6.0%) | 0.21 | 10 (4.1%) | 7 (2.8%) | <0.1 |
| **TRPG, mmHg**** | 24 [20 – 30] | 27 [22 – 33] | 0.32 | 25 [21 – 31] | 25 [22 – 32] | <0.1 |
| **LVM index, g/m^2^*** | 127.9 (124.8 – 131.1) | 126.9 (125.1 – 128.6) | 0.03 | 126.4 (122.2 – 130.6) | 125.7 (121.2 – 130.1) | <0.1 |
| **AV peak velocity, m/s*** | 4.6 (4.6 – 4.7) | 4.5 (4.4 – 4.5) | 0.26 | 4.6 (4.5 – 4.7) | 4.6 (4.5 – 4.7) | <0.1 |
| **AV mean PG, mmHg*** | 51.5 (50.0 – 53.1) | 47.6 (46.7 – 48.4) | 0.23 | 50.2 (48.0 – 52.4) | 51.2 (49.0 – 53.3) | <0.1 |

*Following a normal distribution. Values are means (95% confidence intervals), analyzed by Student’s t-test.

**Not following a normal distribution. Values are median (Inter-quartile-range), analyzed by the Mann–Whitney U test.

SAVR, surgical aortic valve replacement; TAVR, transcatheter aortic valve replacement; SMD: Standardized mean difference; BSA, body surface area; BMI, body mass index; STS-PROM; Society of Thoracic Surgery-Predicted Risk of Mortality, CKD, chronic kidney disease; PMI, pacemaker implantation; OMI, old myocardial infarction; PAD, peripheral artery disease; BAV, bicuspid aortic valve; LVDd, left ventricular diastolic diameter; LVDs, left ventricular systolic diameter; LADi, left atrium diameter index; LVEF, left ventricular ejection fraction; AI, aortic insufficiency; MR, mitral regurgitation; TR, tricuspid regurgitation; TRPG, tricuspid regurgitant pressure gradient; LVM, left ventricular mass; AV, aortic valve; PG, pressure gradient.

**Supplemental Table 3: Postoperative variables in propensity-matched patients**

|  | **Propensity-matched patients** | |  |  |
| --- | --- | --- | --- | --- |
| **Variables** | **SAVR**  **(n=247)** | **TAVR**  **(n=247)** | **P-value** | **SMD** |
| **Implanted valve** |  |  |  |  |
| **Bioprosthetic valve, n** | 240 (97.2%) | - | - | - |
| **Mechanical valve, n** | 7 (2.8%) | - | - | - |
| **Balloon-expandable THV, n** | - | 150 (60.7%) | - | - |
| **Self-expandable THV, n** | - | 97 (39.3%) | - | - |
| **Operative time, min**** | 242 [215 – 288] | 67 [56 – 84] | <0.01 | 3.38 |
| **Hospital stay, days**** | 19[15 – 25] | 7 [5 – 9] | <0.01 | 1.36 |
| **Post echocardiography** |  |  |  |  |
| **LVDd, mm*** | 43.3 (42.6 – 44.0) | 45.6 (44.8 – 46.4) | <0.01 | 0.39 |
| **LVDs, mm*** | 28.6 (27.9 – 29.3) | 29.2 (28.3 – 30.0) | 0.31 | 0.09 |
| **LADi, mm/m^2^*** | 26.5 (26.0 – 27.1) | 28.1 (27.5 – 28.7) | <0.01 | 0.35 |
| **LVEF, %**** | 64 [58 – 70] | 68 [61 – 73] | <0.01 | 0.31 |
| **LVM index, g/m^2*^** | 110.6 (106.6 – 114.5) | 120.7 (116.3 – 125.2) | <0.01 | 0.31 |
| **AV peak velocity, m/s*** | 2.4 (2.3 – 2.4) | 2.2 (2.2 – 2.3) | <0.01 | 0.25 |
| **AV mean PG, mmHg*** | 12.1 (11.5 – 12.6) | 11.1 (10.4 – 11.7) | 0.02 | 0.22 |
| **PVL ≥ mild, n** | 5 (2.0%) | 89 (36.0%) | <0.05†† | 0.87 |
| **PPM ≥ moderate, n** | 66 (26.7%) | 45 (18.2%) | <0.05† | 0.21 |

*Following a normal distribution. Values are means (95% confidence intervals), analyzed by Student’s t-test.

**Not following a normal distribution. Values are median (Inter-quartile-range), analyzed by the Mann–Whitney U test.

†Values are analyzed by χ^2^ test.

††Values are analyzed by Fisher’s exact test.

SAVR, surgical aortic valve replacement; TAVR, transcatheter aortic valve replacement; SMD, Standardized mean difference; THV, transcatheter heart valve; LVDd, left ventricular diastolic diameter; LVDs, left ventricular systolic diameter; LADi, left atrium diameter index; LVEF, left ventricular ejection fraction; LVM, left ventricular mass; AV, aortic valve; PG, pressure gradient; PVL, paravalvular leakage; PPM, prosthesis-patients mismatch.

**Supplemental Table 4. Results of multivariable analysis with or without PVL.**

|  | **Multivariable model without PVL** |  | **Multivariable model with PVL** |  |
| --- | --- | --- | --- | --- |
| **Variables** | OR for LVM regression from baseline to 1-year (95% CI) | **P-value** | OR for LVM regression from baseline to 1-year (95% CI) | **P-value** |
| **Age, years** | 1.01 (0.98 – 1.04) | 0.50 | 1.01(0.98 – 1.04) | 0.39 |
| **Female, n** | 0.84 (0.62 – 1.15) | 0.28 | 0.81 (0.59 – 1.11) | 0.19 |
| **STS-PROM, %** | 0.98 (0.94 – 1.03) | 0.43 | 0.98 (0.94 – 1.03) | 0.41 |
| **Diabetes mellitus, n** | 1.06 (0.77 – 1.47) | 0.71 | 1.03 (0.74 – 1.43) | 0.86 |
| **Atrial fibrillation, n** | 1.30 (0.85 – 1.99) | 0.22 | 1.25 (0.82 – 1.92) | 0.30 |
| **PAD, n** | 1.06 (0.64 – 1.73) | 0.83 | 1.09 (0.66 – 1.79) | 0.75 |
| **BAV, n** | 1.08 (0.55 – 2.09) | 0.83 | 1.10 (056 – 2.14) | 0.79 |
| **Creatinine, mg/dl** | 0.93 (0.84 – 1.02) | 0.14 | 0.92 (0.83 – 1.02) | 0.14 |
| **LVDd, mm** | 1.07 (0.99 – 1.14) | 0.06 | 1.06 (0.99 – 1.13) | 0.09 |
| **LVDs, mm** | 0.87 (0.79 – 0.95) | <0.01 | 0.87 (0.79 – 0.95) | <0.01 |
| **LADi, mm/m^2^** | 0.97 (0.94 – 0.99) | 0.04 | 0.97 (0.94 – 0.99) | 0.04 |
| **LVEF, %** | 0.97 (0.94 – 1.00) | 0.05 | 0.97 (0.94 – 1.00) | 0.05 |
| **AI grade** | 0.96 (0.82 – 1.13) | 0.62 | 0.98 (0.84 – 1.16) | 0.83 |
| **TRPG, mmHg** | 1.01 (0.99 – 1.03) | 0.27 | 1.01 (0.99 – 1.02) | 0.37 |
| **LV mass index, g/m^2^** | 1.04 (1.03 – 1.05) | <0.01 | 1.04 (1.03 – 1.05) | <0.01 |
| **AV peak velocity, m/s** | 1.12 (0.64 – 1.95) | 0.70 | 1.08 (0.61 – 1.89) | 0.79 |
| **AV mean PG, mmHg** | 1.01 (0.98 – 1.03) | 0.65 | 1.00 (0.98 – 1.03) | 0.54 |
| **SAVR, n** | **3.00 (1.74 – 5.13)** | **<0.01** | **2.54 (1.46 – 4.43)** | **<0.01** |
| **PVL ≥ mild, n** | - | - | 0.61 (0.44 – 0.84) | <0.01 |
| **PPM ≥ moderate, n** | 1.27 (0.84 – 1.93) | 0.26 | 1.28 (0.84 – 1.96) | 0.24 |
| **New permanent PMI, n** | 0.76 (0.48 – 1.21) | 0.26 | 0.77 (0.49 – 1.23) | 0.28 |

OR, Odds ratio; LVM, left ventricular mass; CI, confidence interval; STS-PROM; Society of Thoracic Surgery-Predicted Risk of Mortality; PAD, peripheral artery disease; BAV, bicuspid aortic valve; LVDd, left ventricular diastolic diameter; LVDs, left ventricular systolic diameter; LADi, left atrium diameter index; LVEF, left ventricular ejection fraction; AI, aortic insufficiency; TRPG, tricuspid regurgitant pressure gradient; AV, aortic valve; PG, pressure gradient; SAVR, surgical aortic valve replacement; PVL, paravalvular leakage; PPM, prosthesis-patients mismatch; PMI, pacemaker implantation.

**Figure Legend**

**Figure 1. Survival curves freedom from all-cause and cardiac related mortality.**

The 5-year survival curves for (A) freedom from all-cause mortality and (B) freedom from cardiac-related mortality. The shading means pointwise 95% confidence intervals. The insets show the results of landmark analysis.

The survival rates at 5-year follow-up were 87.7% in SAVR and 70.6% in TAVR, with the significant difference [HR0.35 (95%CI 0.20–0.63), P < 0.01]. Furthermore, the freedom from cardiac death survival was also significantly higher in the SAVR group [SAVR vs. TAVR; 93.1 vs. 86.4% at 5-year follow-up, HR0.42 (95%CI 0.18–0.95), P=0.03].

SAVR, surgical aortic valve replacement; TAVR, transcatheter aortic valve replacement; HR, Hazard ratio; CI, confidence interval.

**Supplemental Figure 2. MACCE free survival, details and new PMI rate.**

The 5-year event free survival curves for (A) MACCE, (B) aortic valve re-intervention, (C) heart failure rehospitalization, (D) prosthetic valve endocarditis, (E) cerebrovascular accidents, and (F) new PMI. The shading means pointwise 95% confidence intervals. The insets show the results of landmark analysis.

The SAVR group demonstrated higher MACCE and new PMI free survival compared to the TAVR group [SAVR vs. TAVR; MACCE free survival 70.0 vs. 59.6% at 5-year follow-up, HR0.59 (95%CI 0.37–0.95), P=0.03; new PMI free survival 93.4 vs. 88.7% at 5-year follow-up, HR0.42 (95%CI 0.21–0.83), P < 0.01].

MACCE, major adverse cardiovascular and cerebrovascular events; PMI, pacemaker implantation; SAVR, surgical aortic valve replacement; TAVR, transcatheter aortic valve replacement; HR, Hazard ratio; CI, confidence interval

**Supplemental Figure 3. LVM severity at 1-year follow-up by PVL severity.**

Proportion difference in LVH severity at baseline and 1-year follow-up by PVL severity. Much LV reverse remodeling was observed in patients without PVL. Although the difference was not statistically significant, patients with moderate or greater PVL tended to have less LV reverse remodeling compared to those with mild PVL.

LVM, left ventricular mass; LV, left ventricle; PVL, paravalvular leakage.
